# Effect of anti-müllerian hormone on the development and selection of ovarian follicle in hens

**DOI:** 10.1016/j.psj.2020.12.056

**Published:** 2020-12-26

**Authors:** S.J. Huang, L. Purevsuren, F. Jin, Y.P. Zhang, C.Y. Liang, M.Q. Zhu, F. Wang, C.L. Jia, Z.H. Wei

**Affiliations:** ∗ Key Laboratory of Animal Genetics, Breeding and Reproduction of Shaanxi Province, College of Animal Science and Technology, Northwest A&F University, Yangling, Shaanxi 712100, China; † College of Animal Science, Inner Mongolia Agricultural University, Huhhot 010018, China

**Keywords:** chicken, ovarian follicle, anti-müllerian hormone, granulosa cell, follicle selection

## Abstract

To elucidate the role of anti-müllerian hormone (**AMH**) in regulating the development of ovarian follicles in laying hens, the expressions of follicle-stimulating hormone receptor (***FSHR***), AMH receptor type 2 (***AMHR2***), steroidogenic-related genes steroidogenic acute regulatory protein (***STAR***), cytochrome P450 side-chain cleavage (***CYP11A1***), and 3β-hydroxysteroid dehydrogenase (***HSD3B1***) genes were measured from different sized follicles and granulosa cells. The results showed that the expressions of *FSHR* and *AMHR2* genes were higher in small follicles and decreased after follicular selection. Oppositely, the expressions of *STAR*, *CYP11A1,* and *HSD3B1* were significantly increased after follicular selection. It indicated that AMHR2 might mediate AMH suppression in the stimulating effects of follicle-stimulating hormone (**FSH**) on steroidogenic-related genes expression. To make sure the effects of AMH in this process, a total of 40 hens were treated (negative control, sham operation, 150 ng AMH/d or 300 ng AMH/d) for 25 d. We analyzed ovarian morphology, progesterone concentration in blood plasma, and the expressions of steroidogenic genes in ovaries and follicles. The AMH300 group had significantly lower weight of ovary and hierarchical follicles. Egg weight and ovary weight in AMH150 group were higher than those of sham operation and AMH300 groups, so did hierarchical follicles weight. The steroidogenic genes expressions showed an increase in ovarian tissue and the largest follicle of AMH150 and AMH300 groups. However, progesterone level in the blood was reduced by AMH injection with different concentrations. To further verify the above results, granulosa cells from 6 to 8 mm follicles were cultured with AMH (0, 5, 10, 20, 40, or 80 ng/mL). The results revealed that excessive AMH (80 ng/mL) exerted an inhibitory effect on progesterone synthesis and the expressions of *STAR, CYP11A1,* and *HSD3B1*. However, these genes expressions showed a significant increase in 20 ng/mL AMH-treated group. In summary, AMH inhibited the development of prehierarchical follicles in laying hens. The effects of AMH treatment with different concentrations on follicle development showed the follicle was selected by changing FSH responsiveness of prehierarchical follicles.

## Introduction

Follicular selection is an important process of follicular development and has a strong impact on poultry laying performance. This process occurs in the pool of prehierarchical follicles (6–8 mm in diameter), and 1 follicle on a daily or near-daily is selected to become a hierarchical follicle (Johnson, 2015). After follicular selection, the hierarchical follicle can develop to reach full maturation, and granulosa cells from this follicle initiate differentiation. Generally, hierarchical follicles consist of 5 to 6 large yellow preovulatory follicles (9–40 mm in diameter) called F1–F6 according to the size (Johnson, 2012). The ovulation of the largest preovulatory follicle (**F1**) is accompanied each day by the follicular selection. However, the mechanism of follicle selection is yet unknown. The previous researches reported that several members of the transforming growth factor β family could affect follicular selection (Johnson et al., 2008; Johnson, 2012). Anti-müllerian hormone (**AMH**), a member of the transforming growth factor β superfamily, is mainly secreted by granulosa cells and plays an important role in development and selection of follicles in mammalian ovary (Durlinger et al., 2002). There are also studies that show AMH is principally expressed in granulosa layer of chicken ovarian follicles and markedly decreased at the stage of follicular selection (Durlinger et al., 2002; Johnson, 2012). However, it is uncertain whether the molecular mechanism and function of AMH in chicken follicular selection are similar to that in mammals.

In chicken ovary, AMH is localized to the granulosa cells with strongest expression in the preantral class of follicles. Accompanying follicle selection, the granulosa cells initiate differentiation and progesterone (**P4**) secretion (Robinson and Etches, 1986; Gutiérrez et al., 1997). Therefore, P4 can be identified as a biomarker of follicular selection. *In vitro* and *in vivo* experiments in mice suggest that AMH decreases follicle-stimulating hormone (**FSH**) sensitivity of granulosa cells to affect P4 synthesis (Durlinger et al., 2001). Follicle-stimulating hormone stimulates steroidogenic acute regulatory protein (**STAR/*STAR***), cytochrome P450 side-chain cleavage (**P450scc/*CYP11A1***), and 3β-hydroxysteroid dehydrogenase (**3β-HSD/*HSD3B1***) expression in granulosa cells by binding to its receptor (follicle-stimulating hormone receptor, **FSHR**) (Johnson and Bridgham, 2001). At present, it is well shown that these 3 enzymes are involved in P4 synthesis (Yamazaki et al., 2005; Sechman, 2013). First, STAR mediates the transport of cholesterol from the outer membrane of mitochondria to the inner membrane (Johnson and Bridgham, 2001). Afterwards, P450scc catalyzes conversion of cholesterol to pregnenolone, and 3β-HSD catalyzes the conversion of pregnenolone to P4 (Armstrong et al., 1977; Tilly et al., 1991a; Sechman, 2013; Sechman et al., 2014). These processes occur only in selected hierarchical follicle which is sensitive to FSH stimulation.

It has previously been recognized that AMH reduced the rate of follicular development and FSH sensitivity of granulosa cells by binding to its specific primary receptor (anti-müllerian hormone receptor type 2, **AMHR2**) in rodent (Grootegoed et al., 1994; Baarends et al., 1995; Durlinger et al., 1999, 2001; Mishina et al., 1999), which was relieved after follicular selection (Visser and Themmen, 2005). However, mammalian AMH had no biological activity for chickens (di Clemente et al., 1992). Limited by the absence of purified avian AMH, the effect of AMH on FSH sensitivity of granulosa cells and P4 synthesis in hens could not be directly verified. Recently, the development of prokaryotic expression technology has enabled us to obtain purified chicken AMH.

Herein, this study was designed to compare the underlying effect of different concentration of AMH on follicular selection, P4 synthesis, and egg production in hens by intramuscular injection. Meanwhile, we detected the effects of AMH treatment on granulosa cells from 6 to 8 mm prehierarchical follicles.

## Materials and methods

### Experimental Animals and Management

“Jing Hong I Hao” hens, 29 wk of age and laying actively, were used in this study. All hens were raised in individual cages with 15L: 9D, and the room temperature was kept at 18 to 29°C. Hens were fed 3 times per day (7:00, 12:00, 17:00) with free access to water. For each hen, oviposition time was recorded for at least 10 d before they were used in the experiment. Only hens laying sequences of 9 or more eggs were included in the present study. All experimental procedures were approved by the Animal Care and Use committee of Northwest A&F University (Shaanxi, P.R. China).

### Tissue Collection and Granulosa Cells Collection

To collect ovary and granulosa cells, hens were sacrificed by cervical dislocation in 1.5 to 2 h after oviposition in the morning. The entire ovary was placed immediately into 37°C PBS, and then follicles were classified by size, including small white follicles (**SWF**, 1–3.9 mm), large white follicles (**LWF**, 4–4.9 mm), small yellow follicles (**SYF**, 5–8 mm), the smallest hierarchical follicle (**F6**, 8–9 mm), and F1. Ovary, SWF, follicular membranes (from LWF, SYF, F6, F1), and granulosa layers (from SYF, F6, F1) were collected respectively for RNA extraction.

### Intramuscular Injection of AMH

Forty hens were randomly divided into 4 groups (10 birds for each group): 150 ng AMH-treated group (**AMH150**), 300 ng AMH-treated group (**AMH300**) as described previously (Hayes et al., 2016), negative control group (**CON+**), and blank control group (**CON-**). Anti-müllerian hormone (YU BO Biological Technology, China) dissolved in 0.5 mL sterile water was injected into thigh using a 1 mL syringe with a 23-gauge needle per day. All injections were administered into the left and right thigh muscle alternate days at 8:00 PM for 25 successive d. Hens were weighed before the first injection and after the last injection, respectively. The egg production was recorded daily. The egg and yolk of each egg were individually weighed, and yolk index (yolk weight (g)/egg weight (g) × 100%) was calculated. At the end of the experiment period, blood samples were collected in sterile heparin tubes from the wing vein and centrifuged at 2,800 g for 20 min to separate plasma and then stored at -20°C.

### Ovarian and Oviduct Morphology

After last injection, hens were sacrificed by cervical dislocation in 1.5 to 2 h after oviposition in the morning. The oviduct and ovary with follicles were collected and weighed. The weight of hierarchical follicles was measured by 6 categories F1–F6 (Ebeid et al., 2008). The number of SYF was recorded. The ovarian index and oviduct index (ovary weight, oviduct weight (g)/body weight (g) × 100%) were calculated, respectively. Each tissue was snap frozen and stored at -80°C.

### Granulosa Cells of Prehierarchical Follicles Cultures

Prehierarchical follicles were selected by diameter (6–8 mm). Granulosa cells were isolated and cultured as previously described (Gilbert et al., 1977; Lin and Rui, 2010). After removing the connective tissue and yolk, the follicles were overturned and steeped in ice-cold PBS for 2 to 3 min to rapidly isolate the granulosa layers. Granulosa layer was collected in a 15 mL centrifuge tube and digested with 0.2% collagenase at 37°C for 5 to 10 min. After sieving with 200 mesh, granulosa cells were cultured in M199 medium (Gibco, Gaithersburg, MD) plus 2.5% fetal bovine serum (Gibco), 1% Antibiotic-Antimycotic (Gibco), 1% minimum essential medium nonessential amino acid (Sigma, St. Louis, MO) and 1% minimum essential medium Vitamin (Gibco) at 37°C in a humidified 5% CO_2_ atmosphere for 12 h.

Granulosa cells were then subcultured in 6-well plates till a density of approximately 1.2 × 10^6^ cells per well to be treated with different concentration of AMH (0, 5, 10, 20, 40, or 80 pmol/L) (YU BO Biological Technology, Shanghai, China) for 23 h and then stimulated by recombinant human FSH (100 ng/mL) (YU BO Biological Technology) for 3 h. After treatment, the culture media were collected for enzyme-linked immunosorbent assay (**ELISA**), and plated cells were collected for RNA extraction. All samples were stored at −80°C.

### Progesterone Immunoassay

Progesterone concentration in the culture media of granulosa cells of prehierarchical follicles and plasma from hens of AMH injection was quantified using chicken PROG ELISA Kit (Shanghai ZCI BIO technology co., Ltd., Shanghai, China) according to the manufacturer's instructions method. The optical density of every well was tested at 450 nm using a Microplate reader (BioTek Instruments, Inc., Winooski, VT).

### Quantitative Real-Time PCR Analyses

Total RNA from ovaries, follicular membranes, and granulosa cells was extracted using Trizol isolation Reagent (Invitrogen, Carlsbad, CA) according to the manufacturer's instructions. RNA quality and quantity were assessed on a spectrophotometer (Thermo Scientific, Waltham, MA). Total RNA absorption ratios (260/280 nm) ranged from 1.8 to 2.0. Reverse transcription cDNA synthesis reaction was accomplished using the PrimeScript RT reagent Kit with gDNA Eraser (TaKaRa, Shiga, Japan) according to the conditions described by the manufacturer.

Forward and reverse primers directed toward *Gallus gallus FSHR*, *AMHR2*, *STAR*, *CYP11A1*, *HSD3B1* mRNA and *18S rRNA* were described in Table 1. All primers were designed by Primer Premier 6. Real-time PCR was performed in a CFX96 Real-Time PCR Detection System (BIO-RAD, Hercules, CA). Primers and 75 ng cDNA template were added to 15 μL total reaction volume using the TB Green Premix Ex Taq (TaKaRa). Quantification was accomplished by using the 2^-△△Ct^ method.

**Table 1:** Primer pairs for *G. gallus FSHR, AMHR2, STAR, CYP11A1, HSD3B1,* and *18S rRNA*.

Target	Accession No.	Sequence	Position	Product size
*FSHR*	NM_205079.1	5′-TAATGGAACCTGCCTGGATGA-3′ 5′-CTTGTATGTAGACCTCGCTCTTAG-3′	Fwd Rev	184 bp
*AMHR2*	XM_025152701.1	5′-TCCTTGCTGCTGCTGTCCTTCT-3′ 5′-GCTGTCGTTGTCCTGCCTCCAT-3′	Fwd Rev	192 bp
*STAR*	NM_204686.2	5′-CGCTGCCATCTCCTACCAACAC-3′ 5′-CGACATCTCCATCTCGCTGAAGG-3′	Fwd Rev	196 bp
*CYP11A1*	NM_001001756.1	5′-TCCGCCACCTCAACACCAAGA-3′ 5′-CACAAGGAGGCTGAAGAGGATGC-3′	Fwd Rev	158 bp
*HSD3B1*	NM_205118.1	5′-GCCAAAGAGGAGCAAACCAGAG-3′ 5′-TCCAGCAGTAAGCGAACGATCC-3′	Fwd Rev	104 bp
*18S rRNA*	AF173612.1	5′-TAGTTGGTGGAGCGATTTGTCT-3′ 5′-CGGACATCTAAGGGCATCACA-3′	Fwd Rev	169 bp

Abbreviations: *AMHR2*: anti-müllerian hormone receptor type 2; *CYP11A1*: cytochrome P450 side-chain cleavage; *FSHR*: follicle-stimulating hormone receptor; *HSD3B1*: 3β-hydroxysteroid dehydrogenase; *STAR*: stimulates steroidogenic acute regulatory protein.

### Statistical Analysis

Statistical calculation was conducted using the SPSS18.0 software package. Expression pattern of steroidogenic-related genes in follicular development and the effect of AMH on these genes expression in granulosa cells were analyzed by one-way ANOVA followed by Duncan's multiple range test. The effect of AMH injection on hen ovary was analyzed by contrast to detect differences between treatments. Means separation was via 4 nonorthogonal contrasts: contrast 1 was CON- group vs. 3 injected groups, contrast 2 was CON- group vs. CON+ group, contrast 3 was CON+ group vs. AMH150 and AMH300 groups, and contrast 4 was AMH150 group vs. AMH300 group. *P* < 0.05 were considered statistically significant.

## Results

### Expression Pattern of Steroidogenic Related Genes mRNA in the Follicular Development

**Figure 1:**
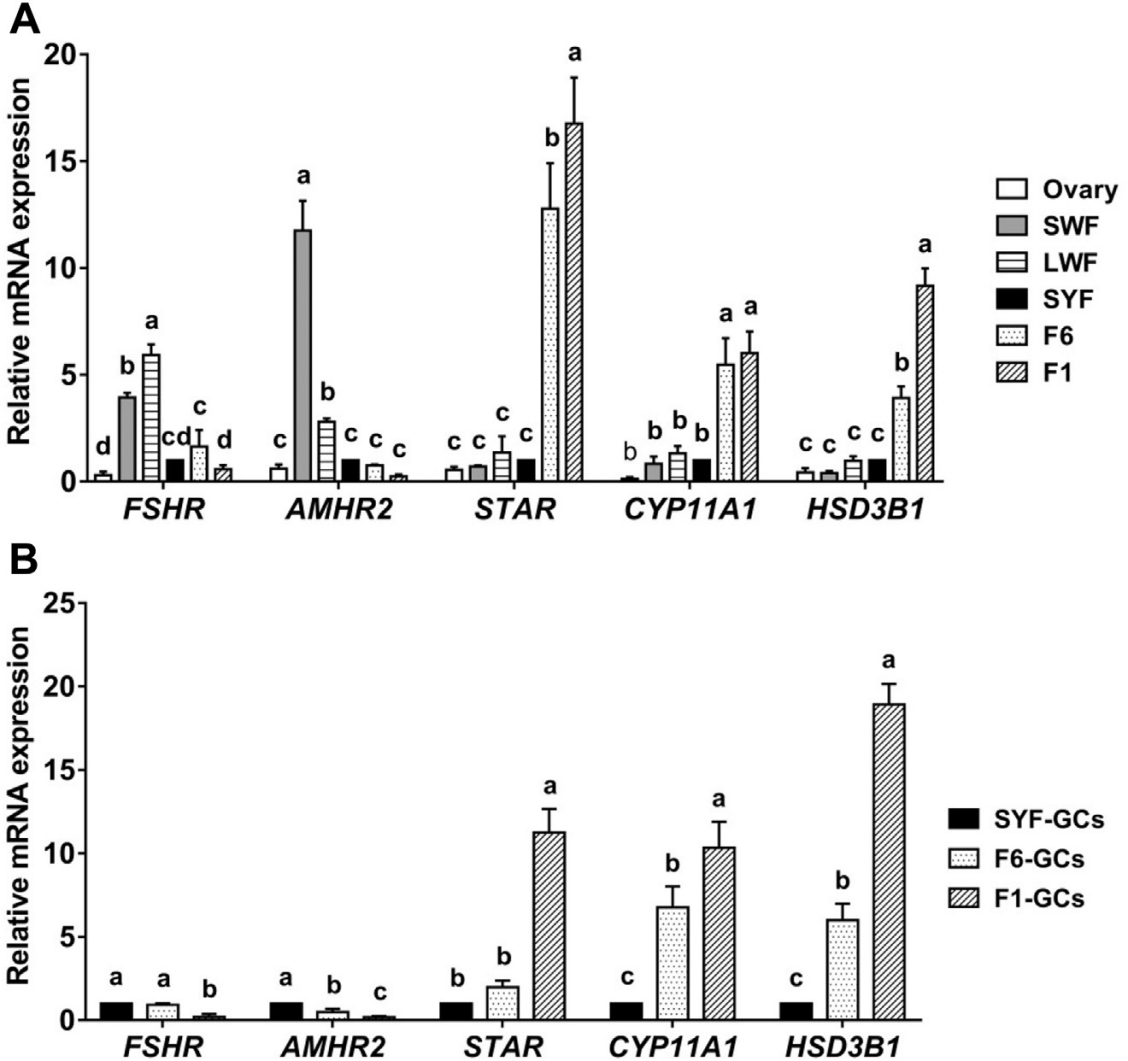
The expression pattern of steroidogenic-related genes mRNA. (A) The related gene expression in ovary and various sized follicles (SWF, LWF, SYF, F6, and F1). (B) The related gene expression in granulosa cells from SYF, F6, and F1. Values were expressed as means ± SD. Values within the same gene with no common lowercase letters (a–c) differ significantly (*P* < 0.05). Abbreviations: *AMHR2,* anti-müllerian hormone receptor type 2; *CYP11A1*, cytochrome P450 side-chain cleavage; F1, the largest hierarchical follicle; F6, the smallest hierarchical follicle; *FSHR,* follicle-stimulating hormone receptor; *HSD3B1,* 3β-hydroxysteroid dehydrogenase; LWF, large white follicles; *STAR,* stimulates steroidogenic acute regulatory protein; SWF, small white follicles; SYF, small yellow follicles.

Real-time PCR results of steroidogenic-related genes expression in different stages of follicular development were showed in Figure 1. The expression of FSHR mRNA was the highest in small follicles (SWF & LWF) and decreased after follicular selection. The expression of *AMHR2* was the highest in SWF and decreased with follicular development. Oppositely, the expressions of *STAR*, *CYP11A1,* and *HSD3B1* mRNA in follicles were increased (*P* < 0.05) after follicular selection (Figure 1A). In addition, the expression pattern of these 5 genes (*FSHR*, *AMHR2*, *STAR*, *CYP11A1,* and *HSD3B1*) in granulosa cells from SYF, F6, and F1 was identical to that of follicles (Figure 1B).

### Effect of AMH on Progesterone Synthesis

**Figure 2:**
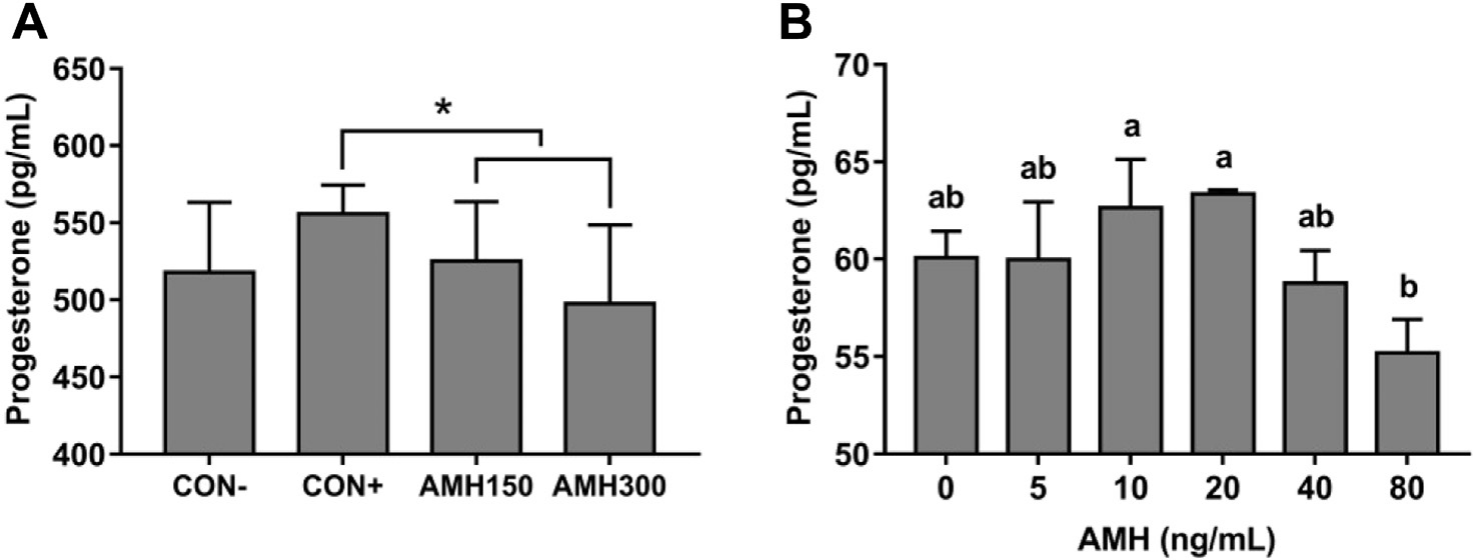
Effect of AMH treatment on progesterone level in granulosa cells culture media and blood plasma. (A) Progesterone level in granulosa cells culture media (n = 3). ∗: *P* < 0.05. (B) Progesterone level in blood plasma (n = 10). Values with no common lowercase letters (a–b) differ significantly (*P* < 0.05). Abbreviations: AMH, anti-müllerian hormone; CON-, no treatment; CON+, sham operation; AMH150, 150 ng AMH/d; AMH300, 300 ng AMH/d.

Progesterone concentration in the granulosa cells culture media and blood was influenced by AMH. There was a significant contrast for the CON+ group vs. AMH150 and AMH300 groups (Figure 2A). Furthermore, P4 concentration was decreased in the media of granulosa cells treated with 80 ng/mL AMH compared with 10 ng/mL and 20 ng/mL AMH. The effect of the other AMH-treated was not shown significant differences for P4 synthesis (Figure 2B). This result showed that excessive AMH might inhibit P4 secretion.

### Regulatory Role of AMH in the Expression of Steroidogenesis Genes mRNA

**Figure 3:**
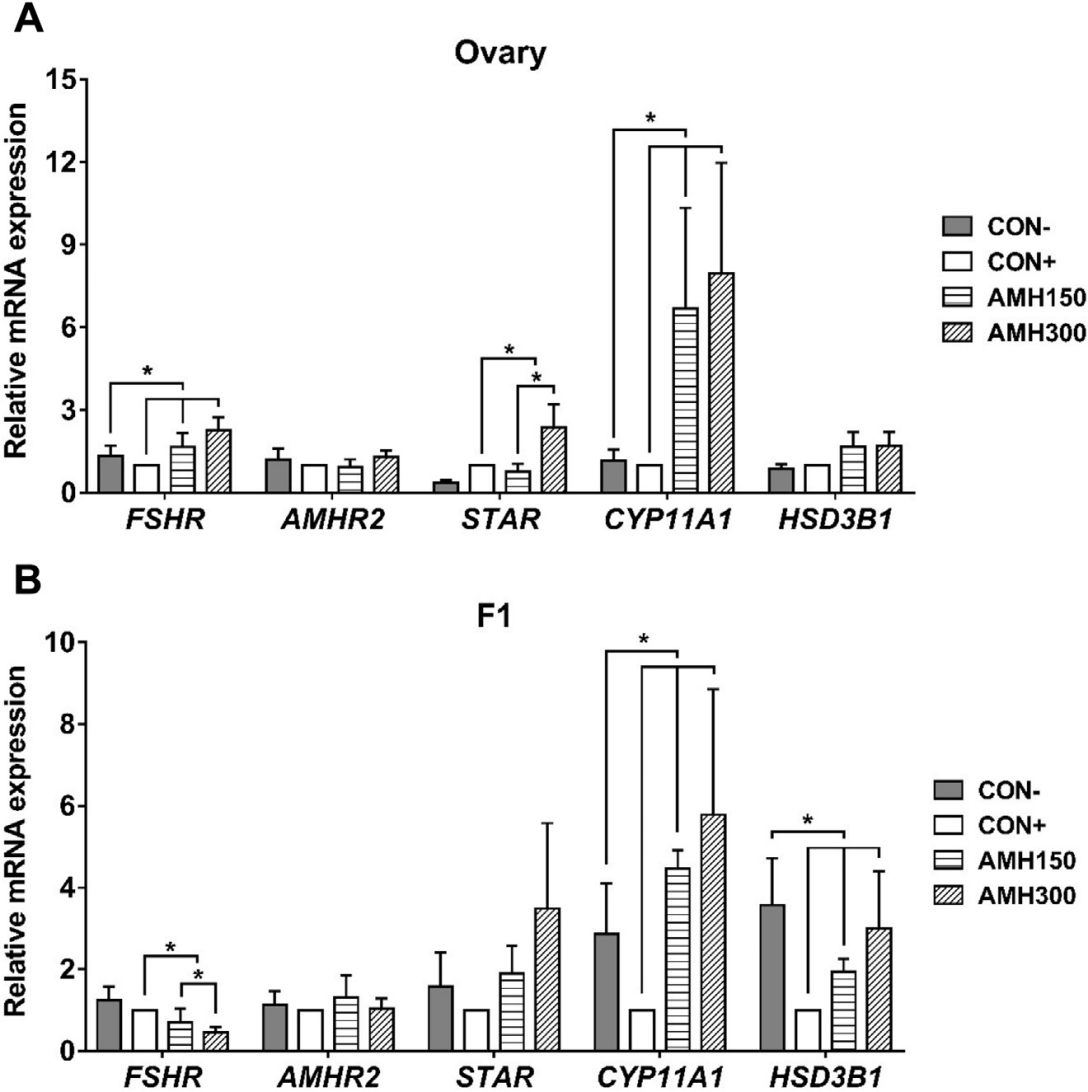
Effect of AMH treatment on expression of genes regulating P4 synthesis in ovary (A) and F1 (B). Contrasts: contrast 1 was CON- group vs. 3 injected groups, contrast 2 was CON- group vs. CON+ group, contrast 3 was CON+ group vs. AMH150 and AMH300 groups, and contrast 4 was AMH150 group vs. AMH300 group. ∗: *P* < 0.05. n = 6. Abbreviations: AMH, anti-müllerian hormone; *AMHR2,* anti-müllerian hormone receptor type 2; *CYP11A1*, cytochrome P450 side-chain cleavage; *FSHR*, follicle-stimulating hormone receptor; *HSD3B1*, 3β-hydroxysteroid dehydrogenase; *STAR*, stimulates steroidogenic acute regulatory protein.

To investigate the function of AMH on P4 synthesis, the expression pattern of steroidogenic gene in ovaries and F1 was analyzed. The expression of *STAR* mRNA in ovary from 2 AMH injection groups was higher than that of CON+ group (*P* < 0.05), and *STAR* gene expression level of AMH300 group was higher than that of AMH150 group (*P* < 0.05). Furthermore, ovarian *FSHR* and *CYP11A1* mRNA expression in 3 injected groups were increased when compared with CON- group (*P* < 0.05; Figure 3A). In the F1 follicle, the expression of *FSHR* mRNA from 2 AMH injection groups was lower than that of CON+ group (*P* < 0.05). Expression of *HSD3B1* mRNA was higher for CON- group than that of other groups (*P* < 0.05). The expression of *CYP11A1* mRNA in the F1 follicle showed a difference (*P* < 0.05) for the CON- group vs. 3 injection groups (Figure 3B). The expression of *AMHR2* mRNA was not influenced by the AMH treatment.

**Figure 4:**
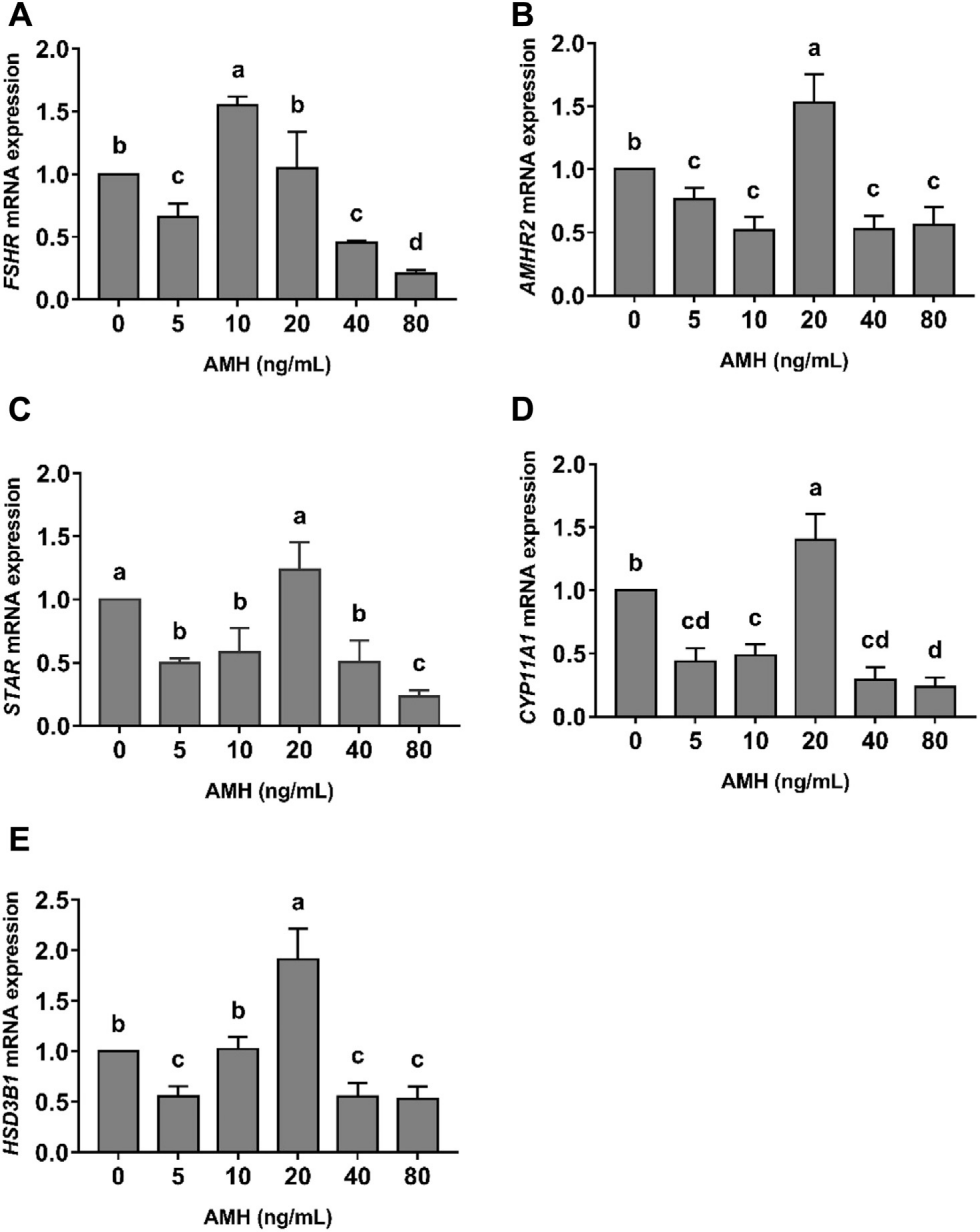
Regulatory role of AMH on critical genes mRNA of steroid production in granulosa cells. (A) Follicle-stimulating hormone receptor (*FSHR*). (B) Anti-müllerian hormone receptor type 2 (*AMHR2*). (C) Stimulates steroidogenic acute regulatory protein (*STAR*). (D) Cytochrome P450 side-chain cleavage (*CYP11A1*). (E) 3β-hydroxysteroid dehydrogenase*H* (*SD3B1*). Values were expressed as means ± SD (*n* = 3 replicate cultures). Values within the same gene with no common lowercase letters (a–d) differ significantly (*P* < 0.05). Abbreviation: AMH, anti-müllerian hormone.

To verify the results of AMH regulating steroidogenic genes *in vivo*, these genes expression in the granulosa cells was analyzed by real-time PCR after AMH and recombinant human FSH treatment. As can be seen from Figure 4, AMH principally inhibited *FSHR* and *AMHR2* mRNA expression, but the expression was promoted for *FSHR* in 10 ng/mL AMH group (*P* < 0.05) and for *AMHR2* in 20 ng/mL AMH group (*P* < 0.05). Similarly, AMH also mainly played an inhibitory role in *STAR*, *CYP11A1,* and *HSD3B1* mRNA expression; however, 20 ng/mL AMH group enhanced the expression of these 3 genes (*P* < 0.05).

### Effect of AMH on Ovary, Oviduct, and Ovarian Follicles

After hens were subjected to daily intramuscular injection with AMH (AMH150 or AMH300) for 25 successive d, and the body weight among the treated groups showed no significant difference. Ovary weight and ovary index from 3 different injection groups were decreased when compared with the CON- group (*P* < 0.05). Ovary weight and ovary index of the CON+ group were lower than CON- group (*P* < 0.05). There was no significant difference in ovary weight and index for CON+ group vs. AMH150 and AMH300 groups. In addition, ovary weight and ovary index of AMH150 group were higher than those of AMH300 group (*P* < 0.05; Table 2). There were no significant contrasts for oviduct weight, oviduct index, and length among 4 groups.

**Table 2:** Effect of AMH treatment on ovary and oviduct characteristics.

Variable	Treatment	Number	Mean ± SD	Contrast^5^			
				1	2	3	4
BW (g)	CON-1	10	1,938.50 ± 38.37	NS	NS	NS	NS
	CON+^2^	10	1,936.40 ± 34.04				
	AMH150^3^	10	1,898.90 ± 24.69				
	AMH300^4^	10	1,923.40 ± 21.74				
Ovary weight (g)	CON-	6	53.62 ± 3.72	∗	∗	NS	∗
	CON+	6	44.5 ± 2.83				
	AMH150	6	48.24 ± 2.18				
	AMH300	6	40.85 ± 2.39				
Ovary index relative to BW (%)	CON-	6	2.71 ± 0.16	∗	∗	NS	∗
	CON+	6	2.29 ± 0.12				
	AMH150	6	2.56 ± 0.12				
	AMH300	6	2.11 ± 0.13				
Oviduct weight (g)	CON-	6	72.56 ± 10.25	NS	NS	NS	NS
	CON+	6	62.99 ± 2.76				
	AMH150	6	60.46 ± 3.06				
	AMH300	6	66.38 ± 2.57				
Oviduct index relative to BW (%)	CON-	6	3.67 ± 0.49	NS	NS	NS	NS
	CON+	6	3.27 ± 0.19				
	AMH150	6	3.22 ± 0.18				
	AMH300	6	3.42 ± 0.16				
Oviduct length (cm)	CON-	6	76.21 ± 4.53	NS	NS	NS	NS
	CON+	6	70.63 ± 3.43				
	AMH150	6	76.41 ± 1.45				
	AMH300	6	74.68 ± 3.26				

Abbreviation: AMH, anti-müllerian hormone.

1 CON- (no treatment).

2 CON+ (sham operation).

3 AMH150 (150 ng AMH/d).

4 AMH300 (300 ng AMH/d).

5 Contrasts: contrast 1 was CON- group vs. 3 injected groups, contrast 2 was CON- group vs. CON+ group, contrast 3 was CON+ group vs. AMH150 and AMH300 groups, and contrast 4 was AMH150 group vs. AMH300 group. Values were expressed as means ± standard error. ∗: *P* < 0.05.

Egg weight of the AMH150 group was higher than that of AMH300 group (*P* < 0.05). There were also differences in egg weight for CON- group vs. CON+ group (*P* < 0.01), CON+ group vs. AMH150 and AMH300 groups (*P* < 0.01), respectively (Table 3). Both yolk weight and yolk index of CON- group were higher than the 3 injection groups (*P* < 0.01), and the result of yolk weight showed a difference (*P* < 0.01) for CON- group vs. CON+ group. There were differences in yolk weight and yolk index for CON+ group vs. AMH150 and AMH300 groups (*P* < 0.05; Table 3). The AMH300 group had lower yolk weight and yolk index compared with AMH150 group (*P* < 0.01). The hierarchical follicles weight showed differences (except F6) for CON- group vs. 3 injected groups (*P* < 0.05), respectively (Table 3). Ovarian hierarchical follicles (F3–F1) weight from CON+ group was lower than those of the CON- group (*P* < 0.05; Table 3). Hierarchical follicles weight (except F2 and F3 follicles) of AMH150 group was higher than those of AMH300 group (*P* < 0.05). There were no significant contrasts for the number of SYF among all treatment groups.

**Table 3:** Effect of AMH treatment on follicle characteristics.

Variable	Treatment	Number	Mean	Contrast^8^			
				1	2	3	4
Egg weight (g)	CON-	10	63.51 ± 0.27	NS	∗∗	∗∗	∗
	CON+	10	61.46 ± 0.33				
	AMH150	10	64.60 ± 0.24				
	AMH300	10	63.56 ± 0.31				
Yolk weight (g)	CON-	10	15.72 ± 0.07	∗∗	∗∗	∗	∗∗
	CON+	10	15.04 ± 0.09				
	AMH150	10	15.75 ± 0.07				
	AMH300	10	14.84 ± 0.09				
Yolk index relative to egg weight (%)	CON-	10	24.78 ± 0.12	∗∗	NS	∗∗	∗∗
	CON+	10	24.50 ± 0.11				
	AMH150	10	24.42 ± 0.12				
	AMH300	10	23.36 ± 0.10				
SYF number^1^	CON-	6	11.16 ± 1.44	NS	NS	NS	NS
	CON+	6	12.66 ± 1.38				
	AMH150	6	10.16 ± 1.04				
	AMH300	6	13.16 ± 1.42				
F1 (g)^2^	CON-	6	14.24 ± 0.40	∗∗	∗∗	NS	∗∗
	CON+	6	12.67 ± 0.43				
	AMH150	6	13.64 ± 0.34				
	AMH300	6	11.95 ± 0.27				
F2 (g)^3^	CON-	6	11.79 ± 0.53	∗	∗	NS	NS
	CON+	6	9.97 ± 0.44				
	AMH150	6	10.56 ± 0.74				
	AMH300	6	9.45 ± 0.50				
F3 (g)^4^	CON-	6	8.94 ± 0.63	∗	∗	NS	NS
	CON+	6	6.89 ± 0.56				
	AMH150	6	8.02 ± 0.45				
	AMH300	6	6.56 ± 0.58				
F4 (g)^5^	CON-	6	5.68 ± 0.52	∗	NS	NS	∗
	CON+	6	4.52 ± 0.45				
	AMH150	6	5.02 ± 0.42				
	AMH300	6	3.58 ± 0.48				
F5 (g)^6^	CON-	6	3.47 ± 0.79	∗	NS	NS	∗
	CON+	6	2.06 ± 0.42				
	AMH150	6	2.49 ± 0.24				
	AMH300	6	1.49 ± 0.26				
F6 (g)^7^	CON-	6	1.45 ± 0.53	NS	NS	NS	∗
	CON+	6	0.93 ± 0.38				
	AMH150	6	1.01 ± 0.19				
	AMH300	6	0.48 ± 0.08				

Abbreviation: AMH, anti-müllerian hormone

∗*P* < 0.05, ∗∗*P* < 0.01.

1 SYF—prehierarchical small yellow follicle (5–10 mm in diameter).

2−7 F1-F6—Hierarchical large yellow follicles (10–35 mm in diameter).

8 Contrasts: contrast 1 was CON- group vs. 3 injected groups, contrast 2 was CON- group vs. CON+ group, contrast 3 was CON+ group vs. AMH150 and AMH300 groups, and contrast 4 was AMH150 group vs. AMH300 group. Values were expressed as means ± standard error.

## Discussion

The development and selection of ovarian follicle have a strong impact on poultry laying performance, so it is important to understand the mechanism of this process. Follicular selection occurs in the pool of prehierarchical follicles. One of the distinguishing features of selected follicles is the onset of P4 synthesis (Johnson et al., 2009). As noted above, the P4 synthesis is regulated by *STAR*, *CYP11A1,* and *HSD3B1*, and these genes expression are stimulated by FSH. What's more, several reports have shown that TGF-β signaling pathway is involved in the initiation of follicular selection for chicken (Grossman et al., 2008; Pellatt et al., 2011; Kim et al., 2013). As a member of TGF-β family, AMH has been suggested to inhibit FSH sensitive of granulosa cells in mouse and human (Brown, 1978; Prapa et al., 2015; Sacchi et al., 2016). However, it is not clear whether AMH can serve the same purpose in poultry as mammals.

In this study, we detected P4 synthesis by ELISA in the culture media of granulosa cells treated with different concentrations of exogenous AMH (0, 5, 10, 20, 40, 80 ng/mL). As far as we know, this is the first work that showed the effect of AMH on P4 synthesis in chicken granulosa cells. As can be seen from Figure 2B, excessive AMH (80 ng/mL) exerted an inhibitory effect on P4 synthesis. For individual level, AMH injection decreased P4 synthesis (*P* < 0.05). The expression of *STAR, CYP11A1,* and *HSD3B1* mRNA was also inhibited in granulosa cells treated with high concentration AMH (Figure 4). These results were consistent with that of Prapa (Prapa et al., 2015) and Sacchi (Sacchi et al., 2016) who suggested that AMH prevented the gonadotropin-induced expression of *CYP19A1* and *CYP11A1* and played a negative role in P4 synthesis by inhibiting STAR protein expression in human granulosa cells. However, it was somewhat surprising in our study that the expression of *STAR, CYP11A1, HSD3B1,* and *AMHR2* showed an increase in 20 ng/mL AMH-treated granulosa cells. In addition, the inhibition of FSHR was relieved in 10 ng/mL and 20 ng/mL AMH-treated groups, which was in according with the previous report in broiler breeder hens (Johnson et al., 2009). They found broiler breeder hens had excessive ovarian follicular development together with an increase in *AMH* mRNA expression after 1 mo *ad libitum* (Johnson et al., 2009). It was also verified in our results that some steroidogenic genes expression showed an increase in ovary and F1 follicle after AMH injection (300 ng/d; Figure 3) (*P* < 0.05). Until now, no data are available about the effect of *in vivo* AMH on the steroidogenic gene expression in chicken. A possible explanation for these was that AMH at an appropriate concentration had a distinct function as inhibiting FSH sensitivity of granulosa cells.

The FSH sensitivity of granulosa cells is related to FSH threshold. In 1978, Brown first defined a certain level of FSH concentrations that was needed for ovarian stimulation as FSH threshold. In mammals, the number of follicles that can develop to reach full maturation depends on the duration of FSH reaching the threshold (Macklon and Fauser, 2001). However, the development of chicken follicles differs from that of mammals. In the pool of prehierarchical follicles, it is approximately that one follicle per day is selected to develop continuously with prioritization, and there is no luteinization after ovulation. Stimulation of FSH is equally for each prehierarchical follicle; therefore, the phenomenon that “only one follicle can be selected a time” cannot be achieved by simply stimulating with an appropriate level of FSH. In addition, de Koning suggested that intraovarian factors were involved in FSH threshold in human (de Koning et al., 2004).

The expression of P4 synthesis-related genes in follicles of different stages was quantified. The results showed that *FSHR* expressed highest in small follicles (SWF, LWF), nonetheless the expression of *STAR*, *CYP11A1,* and *HSD3B1* was deficient in these follicles (Figure 1). These results confirmed that FSH action was suppressed before follicular selection (Tilly et al., 1991b; You et al., 1996; Woods and Johnson, 2005). This study also found that *AMHR2* mRNA was abundant in SWF and LWF. It suggested that strong AMH signal might result in essentially absent of FSH signaling in small follicles. Nevertheless, *FSHR* and *AMHR2* mRNA expression in SYF was relatively low and no difference with F6. The expression of *STAR*, *CYP11A1,* and *HSD3B1* in F6 was remarkably enhanced. Thus, it was suggested that AMH might play a key role in regulating FSH threshold of follicles and was one of the intraovarian factors that caused differences between prehierarchical follicles.

In consistent with *in vitro* results, hens treated with 150 ng/d AMH had higher egg, ovary, and hierarchical follicles weight than that of CON+ and 300 ng/d AMH (*P* < 0.05), but a difference was found between the CON- and CON+ groups (*P* < 0.05), which suggested that the possibility of the stress of injection. However, AMH300 group had the lowest weight of ovary and hierarchical follicles (Tables 2 and 3) (*P* < 0.05). Combined with previous results, it could be hypothesized that ovary and follicular development of hens were affected by different ways depended on AMH concentrations.

In conclusion, the expressions of *FSHR* and *AMHR2* were higher in small follicles and decreased after follicular selection, but the expressions of steroidogenic genes was higher in hierarchical follicles of laying hens. Anti-Müllerian hormone inhibited prehierarchical follicles development by changing FSH threshold to influence follicular selection and P4 synthesis. It indicated when AMH at relatively appropriate concentration could decrease the FSH threshold to promote FSH responsiveness of prehierarchical follicle. Thus, different concentrations of AMH treatment *in vivo* affected ovarian follicle development and steroidogenesis by the different ways in laying hens. This research may be benefit to clarify the mechanism of AMH on the egg laying process in chicken.

## Dislosures

The authors declare that they have no financial and personal relationships with other people or organizations that can inappropriately influence their work, there is no professional or other personal interest of any nature or kind in any product, service and/or company that could be construed as influencing the position presented in, or the review of, the manuscript entitled, “Effect of anti-müllerian hormone on the development and selection of ovarian follicle in hens”.
